# Partial treatment response to capmatinib in *MET*-amplified metastatic intrahepatic cholangiocarcinoma: case report & review of literature

**DOI:** 10.1080/15384047.2022.2029128

**Published:** 2022-02-06

**Authors:** Daniel S Lefler, Marni Brisson Tierno, Babar Bashir

**Affiliations:** a Department of Medical Oncology, Sidney Kimmel Cancer Center, Thomas Jefferson University, Philadelphia, PA, USA; b Foundation Medicine, Cambridge, MA, USA; c Department of Pharmacology & Experimental Therapeutics, Thomas Jefferson University, Philadelphia, PA, USA

**Keywords:** *MET* gene, *met* amplification, cholangiocarcinoma, targeted therapy, capmatinib

## Abstract

Cholangiocarcinoma is a highly morbid gastrointestinal malignancy for which available therapies are limited. Standard of care includes cytotoxic chemotherapies such as gemcitabine, platinum agents, nab-paclitaxel, and fluoropyrimidine analogues. However, tolerability of these regimens varies, and patients who do not tolerate chemotherapy have limited targeted therapies and immunotherapy options. In cholangiocarcinoma, mesenchymal–epithelial transition factor (*MET)* amplification may present an additional opportunity for a targeted therapeutic approach, especially considering emerging data in non-small cell lung cancer. In this case, we present a metastatic cholangiocarcinoma patient with high-level *MET* gene amplification for whom capmatinib, a tyrosine kinase inhibitor with activity against c-MET, provided a partial response after cessation of chemotherapy.

## Introduction

Cholangiocarcinoma (CCA) constitutes 3% of all gastrointestinal tumors and 15% of all liver cancers while carrying a high mortality rate, accounting for 20% of hepatobiliary cancer deaths.^1^ Cholangiocarcinoma is further subdivided based on anatomical site of origin including intrahepatic (10–20%) and extrahepatic, both perihilar (50–60%) and distal (20–30%). The majority of intrahepatic cholangiocarcinoma (iCCA) patients are diagnosed with advanced disease and are not candidates for surgical resection. Though the major focus of treatment involves cytotoxic therapy, performance status may limit the ability for clinicians to offer treatment as the median age at diagnosis is approximately 70 years. CCAs are also highly heterogeneous, often resulting in chemoresistance and poor prognosis.^2^

Comprehensive genomic profiling has become an essential tool for discovering personalized treatment options for advanced cancer patients based on their tumor-specific genomic alterations. Pan-tumor approvals of immunotherapies and targeted therapies for *NTRK* gene fusions have made them attractive options for patients who cannot receive conventional chemotherapy. The KEYNOTE-158 study led to pembrolizumab approval in microsatellite instability high (MSI-H) solid tumors, and activity was successfully demonstrated in MSI-H CCA.^3^ However, these only comprise 1.3% of all CCA cases.^4^ The KEYNOTE-158 study also led to pan-tumor approval of pembrolizumab for patient tumors identified as Tumor Mutational Burden (TMB)-High (TMB ≥ 10 mutations/megabase); however, there were no biliary tract tumors in this study that were found to be TMB-High.^5^ Studies leading to the approval of larotrectinib^6^ and entrectinib^7^ for solid tumors with *NTRK* fusion alterations also included *NTRK* fusion-positive CCA patients, though these cases are rare.

**Figure 1.**
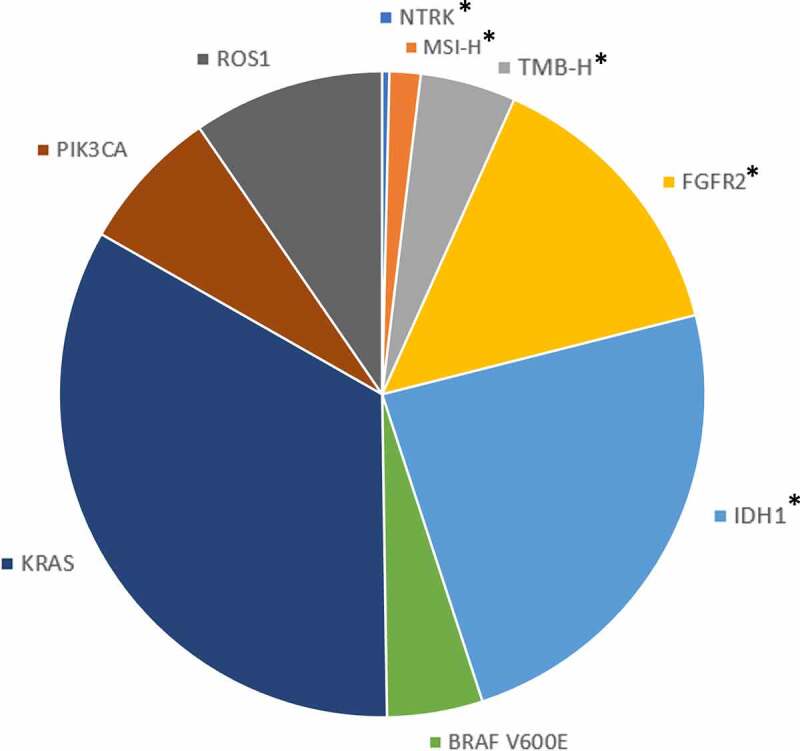
Frequency of actionable and emerging molecular targets & biomarkers in intrahepatic cholangiocarcinoma.

Approximately 50% of CCAs have druggable alterations, allowing for increased use of targeted therapies in CCA.^8^ Currently, guidelines include recommendations for use of targeted agents in patients with unresectable and metastatic disease after progression on primary treatment.^9^ This includes *FGFR2* fusions and rearrangements (found in 9–16% of iCCA cases),^8,10,11^ *IDH1* mutations (16–25%),^8,12^ and *BRAF* V600E mutations (1–6%) (Figure 1).^8,13–15^ There are a variety of additional emerging targets in CCA that may lead to new personalized therapy approaches such as mutations in *KRAS* (22–34% of iCCA) and *PIK3CA* (4–9%) as well as *ROS1* rearrangements (8–9%) (Figure 1).^8,15,16^

(*) indicates NCCN guideline recommended actionable alterations or biomarkers associated with one or more approved targeted therapies for primary or subsequent-line therapy after disease progression for unresectable and metastatic biliary tract cancers. MSI-H: microsatellite instability high; TMB-H: tumor mutational burden high defined as ≥10 mutations (mut)/megabase (Mb).

The mesenchymal-epithelial transition factor (c-MET) is a receptor tyrosine kinase that activates multiple downstream signaling pathways upon hepatocyte growth factor (HGF) ligand binding and dimerization including phosphatidyl inositol 3-kinase (PI3K)/AKT, mitogen activated protein kinase and others.^17^ *MET* gene amplification has been found to be oncogenic and potentially targetable in several solid tumor types including 2% of iCCA cases.^16^ However, there is limited clinical evidence for c-MET inhibition in *MET* amplified CCA patients. A phase II study investigated the multikinase inhibitor cabozantinib in pretreated, unselected patients with advanced CCA resulting in limited activity with high toxicity.^18^ However, one patient considered to have high MET expression (3+ by immunohistochemical (IHC) analysis) experienced a prolonged benefit and duration of treatment. In a phase I study, the c-MET inhibitor tivantinib was given in combination with gemcitabine in patients with advanced or metastatic solid tumors including 8 cases of CCA.^19^ Of the 49 evaluable patients, 39% were strongly positive for MET expression by IHC. Partial response or stable disease was observed in 20% of patients including 1 patient with CCA.

In the following case study, we describe an older male patient with iCCA who was unable to tolerate standard of care cytotoxic therapies. Comprehensive genomic profiling of this patient’s tumor revealed high *MET* gene amplification resulting in the use of capmatinib, a highly potent and selective inhibitor of the MET receptor.

## Case presentation

**Figure 2.**
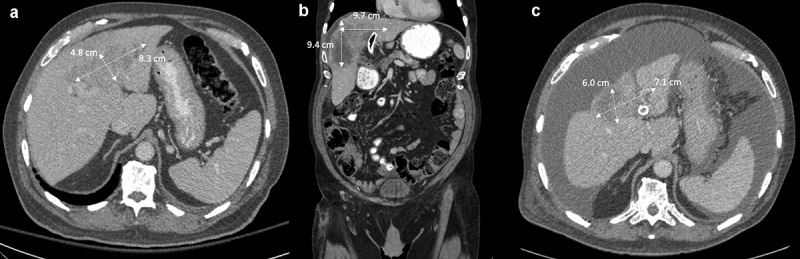
Infiltrative liver mass at various stages of treatment.

A 77-year-old man with a history of spinal stenosis presented to the hospital for planned L4-S1 decompression and fusion. Following surgery, he was noted to have become jaundiced, and liver function testing revealed total and direct bilirubin as 6.4 mg/dL and 5.2 mg/dL, respectively. Cross-sectional imaging was obtained and revealed an infiltrative mass in the liver measuring 8.3 cm x 4.8 cm x 5.9 cm with associated intrahepatic biliary dilatation (Figure 2a). There were scattered, sub-centimeter pulmonary nodules on chest imaging suspicious for metastatic disease.

(A) liver mass at initial diagnosis; (B) after cytotoxic chemotherapy was stopped due to tolerability, prior to initiation of capmatinib; and (C) 3 months after initiation of capmatinib.

He underwent endoscopic retrograde cholangiopancreatography (ERCP), and two stents were deployed into his right and left hepatic ducts, respectively. Biliary brushings and CT-guided liver biopsy revealed a poorly differentiated adenocarcinoma supporting a primary diagnosis of iCCA.

Upon interview in the oncology clinic, the only pertinent family history reported was his mother, with a history of gastric cancer who was deceased by age 59. The option for genetic testing was discussed but the patient opted to defer. Further history revealed that the patient was a veteran of the Vietnam War having been exposed to Agent Orange. He worked in the oil industry later in life. He was a nonsmoker, drank a moderate amount of alcohol (1–2 standard drinks per day), and had never used recreational drugs. After determining that he was not a surgical candidate, he was started on systemic therapy with gemcitabine, cisplatin, and nab-paclitaxel. Patient experienced best objective response of stable disease on this regimen.

Over the course of this regimen, treatment delays and dose reductions were necessary due to the treatment-emergent fatigue and thrombocytopenia, with a nadir of 55,000 platelets per microliter. Ultimately, it was decided that these adverse effects were intolerable, and treatment with cytotoxic chemotherapy was discontinued after 8 months since treatment initiation. This prompted comprehensive genomic profiling using FoundationOne®CDx for consideration of alternate targeted therapies. Genomic profiling results from the tumor tissue revealed immunotherapy biomarkers as microsatellite stable with low TMB (5 mut/Mb), mutations in *TERT* promoter and *TP53*, amplifications of *CDK6, CUL4A*, and *MET*, and equivocal amplifications of *FGF14* and *IRS2*. Of the possible targets, *MET* was considered an appealing option due to the existing literature supporting the use of small molecule tyrosine kinase inhibitors (TKIs) in *MET*-amplified tumors. After discussing available agents, the patient agreed to an empiric trial of capmatinib, a TKI approved for treatment of non-small cell lung cancers (NSCLC) that exhibit an exon 14 skipping mutation in the *MET* gene.

After a four-week treatment holiday to recover from acute toxicities, repeat imaging showed growth of the tumor from 8.9 cm x 7.4 cm x 8.4 cm (while still on chemotherapy, image not shown) to 9.4 cm x 6.9 cm x 9.7 cm (Figure 2b). He was started on capmatinib 400 mg twice daily and tolerated this dose well. Interval imaging 3 months later showed a partial response with decrease in size of the liver mass to 6.0 cm x 3.9 cm x 7.1 cm (Figure 2c). The dose of capmatinib was subsequently decreased to 300 mg twice daily due to anorexia and fatigue, and he continued on therapy for another four months. Two months later, imaging showed slight increase in the size of the mass to 8.5 cm x 6.5 cm x 6.6 cm accompanied by significant tumor necrosis. Approximately, 6 months into therapy with capmatinib, he experienced a precipitous decline in performance status (ECOG 4) due to portal hypertension. He required frequent paracenteses for nonmalignant ascites, and he entered hospice 15 months after his initial diagnosis.

## Discussion

This case represents an example of a *MET*-amplified iCCA that responded to targeted c-MET inhibition. The patient was diagnosed with an inoperable iCCA and was unable to tolerate conventional cytotoxic chemotherapies. Therefore, he was considered for targeted agents after comprehensive genomic profiling of his tumor. Following initiation of the c-MET inhibitor capmatinib, he enjoyed a partial response and nearly six months of improved quality of life.

One of the difficulties in clinical practice is choosing which molecular aberration to target with subsequent-line therapy once standard of care, guideline-directed treatment becomes unfeasible. With the exception of a mutation in *TP53*, most of the genomic alterations detected in this patient’s tumor are not common in iCCA, nor are they associated with approved targeted agents in CCA or other solid tumors. However, the *MET* gene amplification was particularly notable in this case, considering the recent approval of the c-MET tyrosine kinase inhibitor capmatinib for NSCLC patients based on results from the GEOMETRY mono-1 trial.^20^ This phase 2 trial described the use of capmatinib in NSCLC patients whose tumors harbored *MET* exon 14 skipping mutations or were *MET*-amplified. In this study, *MET* gene copy number (GCN) was assessed in tumor tissue by next generation sequencing. In patients with *MET* amplification GCN < 10, ORR was 7–12%. However, those with a GCN of 10 or higher showed an ORR of 29% in previously treated patients and 40% in treatment naïve patients with a median duration of response of around 8 months and median progression free survival (PFS) of just over 4 months.

It is worth noting that detection methods and cutoffs for high-level versus low-level *MET* gene amplification are not standardized. Studies in multiple malignancies, both clinical and preclinical, define the GCN cutoff at varying levels between 4 and 10.^20–22^ In this case, *MET* gene amplification in this patient’s tumor was at a GCN of 23. Despite some categorical uncertainty in the broader medical literature, this GCN was considered to be highly amplified, as defined by the GEOMETRY trial, and was considered an appropriate focus for targeted therapy.

A poorer prognosis is evident in numerous cancers, including CCAs, which exhibit high expression of c-MET.^17,23^ In addition, *MET* mutations and amplifications have been associated with drug resistance in multiple cancer types. Therefore, targeting c-MET is of acute scientific and clinical interest in *MET*-amplified malignancies. Few clinical data exist to support inhibition of c-MET as a standalone therapy, but there is a growing evidence base given the increasing availability of these agents. For example, a 2021 report of results from the AcSé-crizotinib program indicated a role for c-MET inhibition in *MET*-amplified esophageal and gastric cancers. Nine patients with chemotherapy-refractory tumors having ≥6 *MET* copies were treated with crizotinib monotherapy demonstrating an ORR of 33.3%, median PFS of 3.2 months, and overall survival of 8.1 months.^24^

Previous and ongoing studies provide some generalizable evidence for the use of c-MET-directed agents in *MET*-amplified tumors. There are currently an abundance of clinical trials studying selective and nonselective c-MET inhibitors alone or in combination with other therapies across many cancers with *MET* mutations or amplification.^17^ There are currently no ongoing clinical trials specifically targeting c-MET positive CCA, though these patients may be eligible to enter any trial investigating these agents in all solid tumors. With such limited clinical data available, it is hard to draw firm conclusions about the use of these agents in CCA. More clinical trials investigating c-MET specific targeted therapies in CCA patients with *MET*-amplification are warranted. Nevertheless, the foundational science supports the role for c-MET inhibition in *MET* amplified CCA, and this case represents one instance in which a patient benefited from such therapy.

## Conclusions

Comprehensive genomic profiling is crucial for treatment decisions in patients with metastatic cancers, providing clinicians the information to choose appropriate therapeutic options and enroll patients in clinical trials. This is especially important when considering tumor types where standard of care therapies are limited. In this report, we discussed a patient with advanced CCA who was found to have high-level *MET* gene amplification. Because of strong evidence in *MET*-amplified NSCLC in the GEOMETRY mono-1 trial, capmatinib was chosen as subsequent-line therapy after the patient was unable to tolerate cytotoxic chemotherapy. He was found to have a partial response and enjoyed six months of improved quality of life before ultimately experiencing complications of progressive disease and enrolling in hospice.

This case may indicate that c-MET inhibition is a reasonable consideration in patients with *MET*-amplified cholangiocarcinoma in whom cytotoxic therapy is no longer an option. Further, randomized studies should be performed in this population in order to define the role for these agents, which may provide benefit beyond available therapies.
